# Crosstalk between hypertension and diabetes: focusing on pregnancy and offspring. A systematic review

**DOI:** 10.3389/fphys.2025.1519410

**Published:** 2025-04-25

**Authors:** Medina-Hernández Alejandra, Espinosa-Tanguma Ricardo, Donjuán-Loredo Guadalupe

**Affiliations:** Departamento de Fisiología y Biofísica, Facultad de Medicina, Universidad Autónoma de San Luis Potosí, San Luis Potosí, Mexico

**Keywords:** diabetes mellitus, hypertension, insulin resistance, pathophysiological mechanisms, gestational diabetes, hyperglycemia, animal models, humans

## Abstract

**Introduction:**

The coexistence of diabetes mellitus (DM) and hypertension (HT) is very common; both pathologies seem to share different mechanisms such as insulin resistance (IR), endothelial dysfunction, increase in reactive oxygen species (ROS), among others. Furthermore, exposure to hyperglycemia during gestational development has been defined as a risk factor for cardiovascular disease (CVD) in adulthood. However, the mechanisms involved in this “prenatal programming” are still unclear. This review aims to identify the mechanisms involved in the relationship between DM and HT, both in their coexistence and in the development of HT in offspring derived from gestational diabetes (GD). There are no reviews that comprehensively cover both the link between HT and DM as well as the risk factors in mothers with GD and the cardiovascular effects in their offspring.

**Methods:**

A search of published studies reporting HT in offspring of diabetic pregnancies, either in animals or humans, prevalence and pathophysiological mechanisms of binomial hypertension-diabetes (HT/DM), mechanisms, metabolic alterations, DM and HT in pregnancy was done. Inclusion criteria were studies investigating the cardiovascular effects of GD on offspring, studies in animal models or humans, reviews and meta-analyses.

**Results:**

87 studies were included. IR is the main common factor between the presence of DM and the development of HT, in addition to inflammatory processes. Maternal pathology before pregnancy favors the development of diabetes and HT during pregnancy. Animal studies have shown that 100% of the offspring of mothers with GD have HT, mostly after 12 weeks of age. In human studies, there is a significant difference in the blood pressure (BP) levels of the offspring of mothers with gestational hyperglycemia compared with control mothers from the age of 2 years. Several mechanisms such as structural changes in the arterial wall, endoplasmic reticulum (RE) stress, increase in ROS and decrease in nitric oxide (NO) synthesis are proposed as some of the possible culprits.

**Conclusion:**

Current evidence shows that the interaction between DM and HT occurs through mechanisms that they share in their pathogenesis, that is, the presence of one lead to the other and the hyperglycemia to which infants are exposed *in utero* makes them more susceptible to CVD.

## Introduction

DM and HT are two components that coexist worldwide, increasing the risk of CVD and renal disease, as well as the mortality of those who suffer from them. The importance of knowing the bidirectional relationship that exists between both makes it possible to acquire tools for prevention and adequate management of its complications (Sun et al., 2019).

One of the non-modifiable risk factors that implies that these pathologies are present in a large proportion in the world population is genetic load. Mothers who develop it, either before pregnancy or during it, predispose their offspring to the imminent risk of chronic diseases in adulthood.

In this review, we analyze the coexistence of HT and DM, their prevalence, mechanisms involved, risk factors and associated complications specifically in women during pregnancy, and the development of hypertension in offspring, as well as to review the animal models proposed to recreate these conditions and to evaluate the physiological changes proposed as possible responsible.

Our work arises from the lack of reviews that provide an overview of the undeniable link between HT and DM as well as the risk of suffering from these diseases during pregnancy and the adverse effects on their offspring.

The objective of this review is to give a general overview of two highly relevant topics that involve the health of the pregnant mother and her offspring.

## Methodology

### Literature search

**FIGURE 1:**
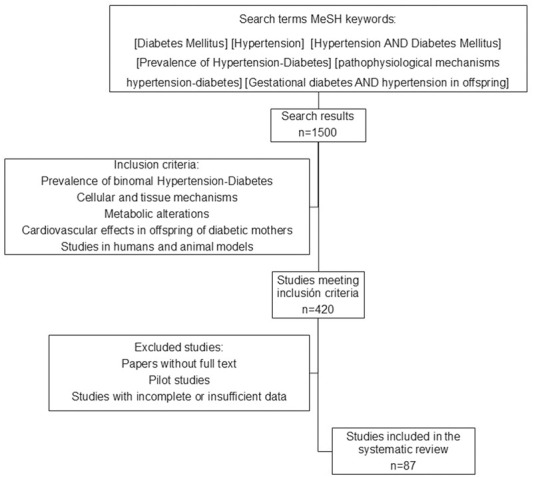
Diabetes/Hypertension binomial. The revised bibliography included the inclusion and exclusion criteria shown in the diagram.

Systematic search of published studies reporting HT and DM. All types of publications, original articles, cohort studies, meta-analyses and reviews were considered. The search was conducted in PubMed (www.ncbi.nlm.nih.gov) and Web of Science (www.webofscience.com) for studies published before February 2025 using Medline subject heading (MeSH) keywords: [Diabetes Mellitus] [Hypertension] [Hypertension AND Diabetes Mellitus] [Prevalence of Hypertension-Diabetes] [pathophysiological mechanisms hypertension-diabetes] [Gestational diabetes AND hypertension in offspring] also, the filter of free full text was applied. The review included animal and human studies. The search was conducted independently by AMH and GEDL from June 2024 to February 2025. Relevant studies were identified from the abstract and selected articles were assessed in full text to determine which would be useful in preparing the review. The data obtained from each article were those that provided valuable information according to the inclusion criteria. Both observational articles were considered, but in the case of animal models it was important that interventions were carried out. For data synthesis, individual summaries and a table were made to synthesize the animal models used to induce GD. (Figure 1).

### Inclusion and exclusion criteria

The inclusion criteria were studies that explored the relationship between HT and DM, their current prevalence, shared pathophysiological mechanisms, the cardiovascular (CV) effects of GD in offspring, studies in animal or human models, reviews and meta-analysis articles. The exclusion criteria were articles without full text, pilot studies, studies with incomplete or insufficient data.

### Data classification

The classification of the selected studies was carried out in four categories: original articles in animals, original articles in humans, reviews and articles that proposed shared mechanisms in pathophysiology, worldwide statistics, associated risk factors in women of reproductive age and pregnant women, signaling pathways involved in the development of HTN/DM and CV pathology of the offspring.

### Data extraction

The following data were extracted from each of the included studies: metabolic pathways, risk factors, epidemiology and prevalence. Study characteristics: first author, published year, study setting, and study design; glucose levels in the mothers, BP measurement and other vascular outcomes of offspring and possible mechanisms proposed for these pathologies.

### Presence of diabetes and hypertension in the world population

According to the World Health Organization (WHO) in 2021, an estimated 537 million adults (ages 20–79) were living with diabetes. The number of people with diabetes is expected to increase to 643 million in 2030 and 783 million in 2045. DM is a leading cause of CVD and mortality (World Health Organizationa).

The WHO estimates that there are 1.28 billion adults aged 30–79 years with HT worldwide and it constitutes the main CV risk factor and is one of the main causes of premature death worldwide; Its prevalence continues to increase (World Health Organization, 2023). Various factors increase the risk of developing HT and include genetic causes, advanced age, alcoholism, overweight or obesity, smoking, unhealthy diets and a sedentary lifestyle (World Health Organization, 2023; World Health Organization, 2019).

Over time, we have seen that CVD is the leading cause of morbidity and mortality in diabetic patients (Gaede et al., 2008). HT occurs in more than 50% of patients with DM; on the other hand, diabetic patients with HT are four times more likely to develop CVD (World Health Organization, 2019). Thus, we have two relationships, one suggesting that DM increases the risk of HT, and another showing the relationship between the initial presence of HT and the subsequent occurrence of DM (Gaede et al., 2008; Song et al., 2016).

### Coexistence between diabetes and hypertension

It is important to mention that the close relationship between DM and HT is also the result of the fact that patients with diagnosed and treated DM maintain stricter BP monitoring than the rest of the population, which favors detection and early initiation of antihypertensive therapy (Sun et al., 2019). In addition, the use of some antihypertensive medications, such as the combination of high-dose thiazides and β-blockers, is associated with an increased risk of DM. Some hypoglycemic medications can induce sodium and fluid retention, which can lead to an increase in BP (Mancia et al., 2006; Zheng et al., 2018).

Many previous studies have shown that HT and DM share common risk factors, including age, gender, smoking, sedentary lifestyle, family history, poor dietary habits, high body mass index (BMI), and waist circumference. Obesity in terms of BMI is the main cause of these diseases (Colosia et al., 2013). They also share pathophysiological pathways that are interrelated and may even lead to a vicious cycle, which we will focus on later (Petrie et al., 2018).

It is still unclear whether the increased risk resulting from the coexistence of DM, and HT is due to a simple combination or to their interactions; nevertheless, their coexistence is no coincidence (Yu and Liu, 2004). Some studies have suggested that this bidirectional relationship has a genetic component, with genetic DM being the one that increases the risk of HT. They even suggest the presence of accelerated arterial stiffness resulting from DM, which is associated with a greater increase. of heart rate (HR) and BP specifically systolic (SBP).

### Pathophysiological mechanisms shared

**FIGURE 2:**
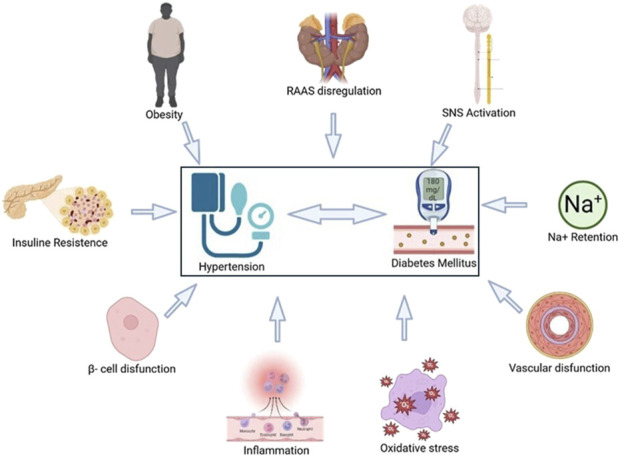
Physiopathological mechanisms involved in the co-development of hypertension (HT) and diabetes mellitus (DM). The combination of these factors deteriorates homeostasis and allows the relationship between both diseases, establishing them as a multifactorial phenomenon that is accompanied by complications that reduce people’s quality of life. Created by Biorender.

This binomial shares several pathogenic pathways such as obesity, IR, pancreatic β-cell dysfunction, inflammation, oxidative stress, vascular dysfunction, sodium retention, sympathetic excitation, activation of the renin-angiotensin-aldosterone system (RAAS), and renal damage (Figure 2) (Lip et al., 2016).

Evidence suggests that the association between obesity and DM in hypertensive patients, as well as IR in obesity and HT, share not one but several pathophysiological factors (Lastra et al., 2014). One is the presence of higher HR, which is an important marker of CV risk in DM, something we also observe in obesity (Fox et al., 2007); this is a measure of sympathovagal balance (Hillis et al., 2012). HR may be an indicator of impaired sympathetic nervous system (SNS) function associated with DM; although HR alone does not necessarily confer risk of HT, it is the sympathovagal imbalance that causes diabetic patients to develop HT (Liu et al., 2019).

The combination of both chronic diseases has adverse effects on left ventricular structure, myocardial dysfunction, and arterial stiffness. Both DM and HT are risk factors for the development of atherosclerosis and are essential components of the deterioration and exacerbation of endothelial and smooth muscle function. Their combination promotes endothelial cell dysfunction by generating oxygen-derived free radicals that damage endothelial function. They also promote monocyte adhesion to endothelial cells, thereby increasing vascular superoxide production and monocyte chemoattractant protein-1 expression, leading to atherosclerosis and subsequent cerebrovascular/CV disease (Wang et al., 2021).

A widely recognized mechanism is inflammation, which is the main pathological process in many diseases (Ning et al., 2021). Low-grade inflammation induced by IR is the main mechanism explaining the development of endothelial dysfunction and metabolic abnormalities in obesity (Carrizzo et al., 2018).

In inflammatory metabolic disorders proinflammatory cytokines (tumor necrosis factor alpha (TNF-α), interleukin-6 (IL-6) and monocyte chemoattractant protein-1) can alter insulin metabolic signaling and reduce insulin-mediated nitric oxide (NO) production, leading to arterial stiffness and HT. Furthermore, systemic and tissue inflammation are strongly related to abdominal obesity (Jia et al., 2017); since macrophage infiltration is an important driver of adipose tissue inflammation and metabolic disorders associated with HT. In the setting of obesity, IR, and DM, adipokines from perivascular adipose tissue contribute to vascular IR and impaired relaxation (Thanassoulis et al., 2012).

The current literature strongly supports the idea that HT and DM share another common pathophysiological mechanism: IR; this is defined as a biological effect that is lower than expected in response to a given concentration of the hormone and this plays a fundamental role in the pathogenesis of DM (Cheung and Li, 2012). In untreated fasting hypertensive patients, postprandial insulin levels are higher than in normotensive patients; similarly, it has been documented that rats with genetic HT such as DAHL or SHR strains show IR and hyperinsulinemia (Ferrannini et al., 1987). And in rats fed for 6 months with a high-calorie diet, they show that the increase in BP and the development of left ventricular hypertrophy are associated with hyperinsulinemia or hyperglycemia (Mazzone et al., 2018).

It is important to note that insulin is a hormone that does not exclusively affect glucose metabolism, but also affects lipid and protein metabolism, ion and amino acid transport, cell cycle, proliferation and differentiation, and NO synthesis (Scherrer et al., 1994).

IR also contributes to elevated BP through other mechanisms, including increased angiotensin II (AngII) and aldosterone activity (Steinberg et al., 1996; Fukuda et al., 2001), and increased SNS activity (Lembo et al., 1992); in this sense in hypertensive patients, the SNS response to insulin has increased (Lembo et al., 1992). This increase in SNS tone impairs the vasorelaxant effect of insulin and contributes to elevated BP (Lembo et al., 1992). It also induces stimulation of β-adrenergic receptors that promote IR (Morisco et al., 2005).

Likewise, RAAS deregulation plays a fundamental role in the development of IR and HT; thus, prevention of DM in patients with HT requires therapeutic inhibition of the RAAS with angiotensin type 1 receptor (AT-1R) antagonists or angiotensin-converting enzyme (ACE) inhibitors (Borghi et al., 2015; Yusuf et al., 2001; Julius et al., 2004). *In vivo* and *in vitro* studies have shown that IR and hyperglycemia induce systemic activation of the RAAS associated with increased vascular resistance and BP. Ang II and aldosterone decreased activation of endothelial nitric oxide synthase (eNOS) and reduced NO-mediated vasodilation (Miller et al., 1996). Hyperinsulinemia associated with insulin resistance stimulates production of the vasoconstrictor endothelin-1 (ET-1) contributes to excessive arterial stiffness, and HT. Hyperinsulinemia and aldosterone have been found to increase endothelial sodium channel activity, leading to arterial stiffness and HT (Miller et al., 1996; Mancusi et al., 2020).

The existence of a common genetic pathway for HT and IR is supported by the altered glucose metabolism found in normotensive offspring of hypertensive patients (Grunfeld et al., 1994). In addition, specific genetic abnormalities have been identified in individuals with combinations of IR, obesity, dyslipidemia, and HT, such as mutations in β3-adrenergic receptors (Cheng et al., 2001) or deficiency of CD36, a known fatty acid transporter involved in predisposition to IR and HT (Miyaoka et al., 2001). The relationship between IR and HT is a complex and multifactorial phenomenon involving both genetic and environmental factors, hence the importance of studying both those who suffer from it and their offspring.

### How can the health of a pregnant women can be affected by diabetes and hypertension?

Of particular interest is the Women’s Health Study (Conen et al., 2007), which showed that in healthy middle-aged women, progressive elevation of BP is a strong and independent predictor of DM. Similarly, the Monitoring Trends and Determinants on CVD/Cooperative Health Research study from the Augsburg region (Meisinger et al., 2008) showed that established HT in women significantly increase the risk of DM, and glucose intolerance and DM have a higher incidence of HT than men (Meisinger et al., 2008).

Hypertensive disorders of pregnancy are common, affecting approximately 5%–9% of normal pregnancies. Given the increasing prevalence of HT, obesity, and DM in women of childbearing age and the societal trend toward advanced maternal age, published rates may underestimate recent incidence. These disorders are a major cause of maternal and neonatal morbidity and mortality. The diagnosis and management of HT in pregnancy requires attention to the maternal and fetal effects of the disease and its treatment (Hutcheon et al., 2011).

Hemodynamically, there are significant physiological changes in pregnancy and a reduction in systemic vascular resistance, RAAS is activated early in pregnancy without affecting BP. A decrease in BP influenced by the vasodilator relaxin, ovarian hormone, NO signaling and the vasodilatory state (Bramham et al., 2014; Cunningham et al., 2018).

Controlled chronic HT is usually well tolerated during pregnancy with minimal maternal and fetal complications; however, women with uncontrolled BP are at increased risk for poor obstetric outcomes and target organ damage. Gestational HT, or pre-eclampsia (PE), is the development of newly elevated BP after 20 weeks of gestation without evidence of maternal organ dysfunction; this should usually be resolved by 12 weeks *postpartum* (van Oostwaard et al., 2015; Magnussen et al., 2009).

Gestational diabetes (GD) and PE are common complications of pregnancy with similar risk factors, including obesity, advanced age, and multiple gestation. In the United States (US), GD was diagnosed in 7.8 per 100 births, an increase of 30% since 2016. The Pan-American Health Organization reports that GD is diagnosed in one–two of every 10 pregnancies in the Americas, but cases continue to increase [49 50].

GD is defined as a blood glucose level higher than normal but lower than that which justifies a diagnosis of diabetes that appears during pregnancy (WHO) (Diabetes; WHO, 2013). In pregnant women there are three tests to establish the diagnosis of GD: casual blood glucose greater than 200 mg/dL and classic symptoms of diabetes; fasting glucose greater than 126 mg/dL on two or more occasions; and serum or plasma glucose concentration 1 hour after ingesting 50 g of glucose greater than 140 mg/dL (corroborated with an oral glucose tolerance curve with values ​​greater than 180 mg/dL) (Org.mx, 2013). Increased insulin resistance, pancreatic β-cell dysfunction are the main pathogenesis of GD, which may be present before pregnancy, especially in populations of women with obesity (Marschner et al., 2023).

Studies have shown that the incidence of PE increases significantly in the presence of GD. PE alone is the leading cause of maternal morbidity and fetal morbidity/mortality. However, GD complicated by PE further increases perinatal adverse events as well as the future risk of CVD and metabolic syndrome (MetS) in both the mother and offspring (for example, offspring BMI increases steadily over time); therefore, identifying factors associated with the occurrence of PE in women with GD, especially those that are controllable, is important to improve pregnancy outcomes (Yang and Wu, 2022; McIntyre et al., 2019).

Furthermore, the pathophysiological processes in both GD and PE include oxidative stress, release of pro-inflammatory factors, and vascular endothelial dysfunction, which increase the risk of future maternal diabetes and CVD, as well as neonatal complications (McIntyre et al., 2019; Phoswa and Khaliq, 2021).

The mechanisms of the association between GD and PE are not entirely clear. The pathophysiological process of PE involves placental ischemia, oxidative stress, increased levels of antiangiogenic factors in placenta that cause inflammation and vascular endothelial dysfunction (Rana et al., 2019). On the other hand, GD can induce trophoblast inflammation, increase oxidative stress through a variety of pathways, including the formation of advanced glycation end products (AGEs), increased production of reactive oxygen species resulting in a decrease in NO levels which may promote the occurrence of PE. In addition, an increase in proinflammatory cytokines associated with endothelial dysfunction and PE has been observed in women with GD. Even TNF-α, IL-6 and C-reactive protein (CRP) have been suggested as independent risk factors for PE in women with GD (Yang and Wu, 2022).

Another important factor influencing the development of PE in women with GD is obesity, which is one of the world’s major health problems. According to global estimates, the incidence of obesity has doubled in the last 4 decades and more than 30% of women are obese (Yao et al., 2020). This epidemic increase in obesity is also reflected in a higher prevalence of maternal obesity. In the US, more than 50% of pregnant women are considered obese, while in Europe, the number of women considered overweight and obese during pregnancy has also increased significantly, reaching rates of 30%–37% (Aubry et al., 2019). Obesity has been shown to affect maternal and infant health by increasing the risk of adverse perinatal outcomes (Yao et al., 2020; Plows et al., 2018).

### Effects of gestational diabetes and hypertension on offspring

In the literature, we found that the coexistence of hypertension and gestational diabetes (GD) in mothers may disappear after birth, in offspring, it can cause complications throughout their lives and was associated with adverse neonatal outcomes, including small for gestational age at delivery and/or small for gestational age at birth, perinatal mortality, fetal macrosomia, malformations, congenital disease, respiratory distress syndrome, low APGAR, neonatal hypoglycemia and, in general, a higher incidence of offspring with metabolic disease in adulthood (Lewandowska et al., 2020). In turn, in the long term, a higher prevalence of obesity, insulin resistance, glucose intolerance, and vascular complications has been observed compared to offspring of mothers with normal blood glucose levels (Gob.mx; Avgil Tsadok et al., 2011).

The predisposition that descendants of mothers with GD have to suffer from CVD in adulthood, mainly HT, is gathering more evidence every day, presenting higher pressure values ​​from the age of 3 years, this difference becoming increasingly greater with the passing of the time in their life concerning the offspring of mothers with normal blood glucose levels (Wright et al., 2009; Cho et al., 2000; Leybovitz-Haleluya et al., 2018; Yu et al., 2019) However, the mechanism by which this “metabolic programming” occurs is still not fully understood.

### Animal models

**FIGURE 3:**
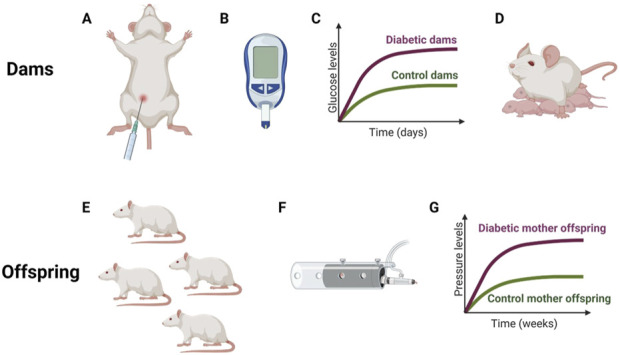
General procedure in mothers and their offspring **(A)**. Intraperitoneal injection of streptozocin at day 0 of gestation. **(B)**. Glucometer used to check glucose levels. **(C)**. Hypothetical graph showing glucose differences throughout pregnancy between two groups of mothers. **(D)**. Mother with their newborn rats. **(E)**. Adult offspring. **(F)**. Noninvasive system used to measure blood pressure in rodents. **(G)**. Hypothetical graph showing pressure differences between the offspring of control and diabetic mothers. Created by Biorender.

The use of animal models has allowed us to further study the mechanisms involved in certain pathologies. In this case, the GD model has been generated in rats to then evaluate the CV effects in their offspring. The model used in general to develop diabetes in mothers is by intraperitoneally injecting streptozotocin (STZ), a drug that destroys the beta cells of the pancreas, so the rats stop producing insulin and this leads them to have high glucose levels. The values achieved range from 15 to 20 mM, that is, between 250 and 350 mg/dL. The STZ injection is done on day 0 of pregnancy, which is when the sperm plug is observed. High glucose concentrations are reached approximately after 3 days but are monitored throughout the pregnancy to corroborate the maintenance of hyperglycemia. Once the offspring are born, they remain with their mothers until weaning and that is when the evaluation of the BP values and any other parameters can begin. As shown in Figure 3 the device used to measure glucose levels in rats is the same one used in humans, but the one used to measure BP is one designed specifically for rodents. Three articles of the five selected in this review explain the animal model, refer to the onset of HT in the offspring of diabetic dams at 12 weeks of age (Luo et al., 2022; Yan et al., 2014; Luo et al., 2018) while another two refer that these values were reached after 6 and 18 months respectively (Dib et al., 2018; Nehiri et al., 2008). To perform molecular and/or tissue-specific tests on the offspring of diabetic mothers and control mothers, they are allowed to continue growing until a certain age and then the sacrifice is carried out.

Different proposed mechanisms could be involved in this development of HT in the children of mothers with GD. Lou et al., in two independent publications, report having observed an increase in ROS and ER stress; a decrease in the synthesis of NO; impaired diuresis and natriuretic accompanied by an increase in renal protein kinase C (PKC) (Luo et al., 2022; Luo et al., 2018).

The decrease in NO production is directly related to the state of HT since by losing the ability to produce it; the capacity for vasodilation is also lost in response to increases in blood flow and therefore a continuous increase in pressure of the vessel walls which could lead to the thickening of the vessel walls to be able to withstand this increase in pressure which was reported by Dib et al. and Nehiri et al. who refer to structural changes in the walls of blood vessels and the glomeruli, respectively, which could have implications for pressure values (Dib et al., 2018; Nehiri et al., 2008). In the work of Yan et al., they found alterations in the genes of the RAAS, which we know is highly involved in the control of BP, and increased glomerular filtration, which could indicate an early stage of kidney dysfunction (Yan et al., 2014). Table 1 shows a summary of the aforementioned information specifying the rat strains and the dose of STZ used.

**TABLE 1:** Animal models of gestational diabetes using streptozotocin, development of hypertension in offspring and proposed mechanism involved in the development of this cardiovascular disease.

DAMS			OFFSPRING		Ref.
Animal/Strain	Diabetes model	Glucose levels reached	Age of hypertension start	Proposed mechanism	
Rat/Sprague-Dawley	STZ Injection of 35 mg/kg at 0 days of gestation.	17 mM	12 weeks	There is fetal vascular programming where AMPK levels are decreased, which could lead to an increase in RE stress, an increase in ROS, and a decrease in NO synthesis	Luo et al. (2018)
Rat/Wistar	STZ Injection of 25 mg/kg at 0 days of gestation.	15 mM	12 weeks	Fetal programming causes an alteration in the expression of the component genes of the RAAS system. An increased glomerular filtration rate was also found, indicating an early stage of renal dysfunction	Yan et al. (2014)
Rat/Sprague-Dawley	STZ Injection of 35 mg/kg at 0 days of gestation.	18 mM	12 weeks	Impaired diuresis and natriuresis are mediated by D1 receptor, accompanied by increased renal PKC expression and activity	Luo et al. (2018)
Rat/Sprague-Dawley	STZ Injection of 35 mg/kg at 0 days of gestation.	450 mg/dL (25 mM)	18 months	Fetal programming led to structural changes in the vasculature: an increase in area of the medial layer and a decrease in arterial diameter, which could be responsible for the increase in BP.	Luo et al. (2018)
Rat/Sprague-Dawley	STZ Injection of 35 mg/kg at 0 days of gestation.	20 mM	6 months	Glomerular hypertrophy, decreased renal function assessed by altered creatinine filtration values and proteinuria	Dib et al. (2018)

### Human studies

Longitudinal studies in humans are difficult to carry out since it is difficult to maintain contact with patients over the years due to changes in residence, loss of contact or simply because they do not want to continue with the study. Despite this, we have found studies of up to 40 years of follow-up in the offspring of mothers with GD and the prevalence of cardiovascular problems.

In a study done by Tsadok et al. where the offspring of GD and non-diabetic mothers in Jerusalem were followed for 17 years, they found a significant difference in weight, BMI, and systolic and diastolic pressure, all of which were higher in the adolescent children of mothers with GD (Gob.mx). In another work carried out by Cho et al. In an American child population (12 and 13 years old), it was found that children descended from mothers with GD presented higher values of BMI, SBP and mean arterial pressure; as well as elevated values at 2 h of both glucose and insulin in the corresponding resistance tolerance curves (Cho et al., 2000).

Yu et al. did a follow-up on the Danish population born between 1977 and 2016 for up to 40 years. They observed a 29% increased risk of developing CVD early in life if they had been exposed to hyperglycemia in the mother’s womb. Different CVD were evaluated, and it was found that these offspring had a 45% higher risk of heart failure, 78% of HT, 82% of deep brain thrombosis and 91% of pulmonary embolism compared to those from mothers with normal blood glucose levels (Yu et al., 2019).

The population of the Viva Project carried out in Massachusetts was followed 3 years after birth to children born to mothers with glucose intolerance developed during pregnancy and GD as such, here they found a significant difference in SBP values, being 1 mmHg and 4 mmHg higher in these groups compared to controls (Wright et al., 2009). This increase in pressure values ​​may seem very small, however, being at a very young age, it is very relevant since in these cases it is observed that the difference concerning the controls becomes larger with time.

Jun Lu et al. did a study in the city of Tianjin, China where they followed the study population for 6 years and found that children who had been exposed to hyperglycemia in the womb and their mothers had a higher body mass index, had higher SBP values ​​and also a higher prevalence of HT, it should be noted that it is a very young age to present this condition (Lu et al., 2019). In addition to HT, other CV conditions have been found in the offspring of mothers with GD, such as arrhythmia at 18 years of age (Wright et al., 2009) and heart disease at 1 week of birth (Martinez-Garcia et al., 2020).

## Discussion

The prevalence of HT/DM continues to increase throughout the world, so it is of utmost importance to know the binomial present in pregnancy, mechanisms as well as to identify and understand the consequences that these conditions can have on both the mother and the offspring. It has been reported that complications in mothers are normally lost as soon as they give birth; however, the effects on their offspring appear over time.

This review showed a general overview of the coexistence of HT/DM in the general population; but in mothers, GD implies an exposure to hyperglycemia in gestation and increased CV pathologies in the offspring in different stages of their life. In humans, a greater risk of developing HT has been observed from 3 to 40 years of age, where there is already a well-classified development of the disease. In rats, which are the most used animal model for the generation of GD and the evaluation of its effects on the offspring, it has been observed that from 12 weeks they begin to present HT values, structural damage to the blood vessels, and malfunction of the Renal System. This age of 12 weeks in rats extrapolated to humans would represent a child of approximately 8 years (La Rata de Laboratorio - Relacionar Su Edad Con La de Los Humanos, 2022). In most studies, the increase in pressure was observed in SBP, where it has been reported that in adulthood an increase of only 2 mmHg is related to an increase in mortality (National Clinical Guideline Centre UK, 2020).

**FIGURE 4:**
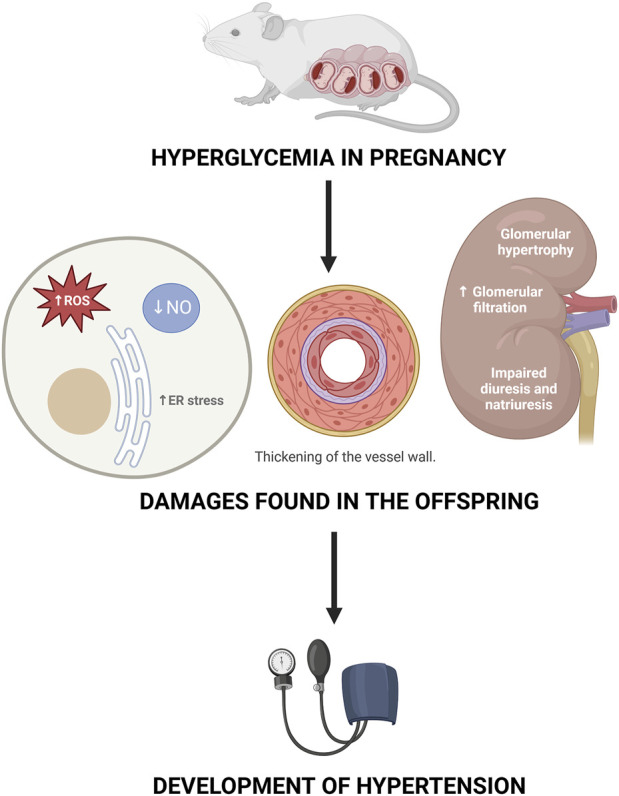
Mechanisms proposed as responsible for the development of hypertension in the offspring of mothers with gestational diabetes.

Different signaling pathways are demonstrated to be involved in this development of HT, such as ER stress, NO synthesis, and generation of ROS, among others (Figure 4). However, it is not known what the timing of these events is, that is, if the disease develops first and then the alterations in these pathways occur or if the disease occurs because these pathways are altered.

Another point to consider is the epigenetic changes that occur in the offspring due to exposure to hyperglycemia in the uterus. Epigenetics refers to changes in gene expression without making modifications to the DNA sequence. Within the epigenetic modulators, we can find DNA methylation, histone modification and microRNAs; all of which affect the protein expression levels of the modified genes. In the case of GD, it has been reported that it can affect methylation patterns and gene expression in offspring, which can lead to complications throughout their lives. (Dłuski et al., 2021). In animal models, it has been found that the epigenetic changes caused by GD can be passed from generation to generation. An example of this is the Dlk1 gene, which is involved in the activation of the insulin/IGF-I signaling pathway and which Jiang et al. found downregulated until the second generation. (Franzago et al., 2019). Speaking specifically of CVD, gene modifications have been found in the offspring of mothers with GD at the level of arteries such as the thoracic aorta, where overexpression of potent vasoconstrictors such as Cyp4f4 and downregulation of the IP gene, involved in vasodilation, have been observed. The modifications in the expression of these genes are an example of what could contribute to the development of HT in the offspring of mothers with GD thanks to the prenatal programming given by hyperglycemia in the intrauterine environment. (Van Huyen et al., 2010).

Glucose, being able to cross the placenta by facilitated diffusion, generates problems in both the pregnant mother and the baby when there is a dysregulation in its concentration, so it is important to mention the measures that can be taken to prevent high levels of it. (Diagnóstico, 2009). In the case of women who are of reproductive age and want to become pregnant, they should try to maintain their normal weight and perform physical activity constantly. (Kitzmiller et al., 1991). A woman considered with high risk of GD present these characteristics: severe obesity, a history of GD in first-degree relatives, GD or glucose intolerance in a previous pregnancy, a history of macrosomic products, or current glycosuria. (American diabetes Association, 2008). In high-risk women, it is recommended to perform glucose screening between 12 and 14 weeks of gestation, in low- and moderate-risk women the screening is performed between 24 and 28 weeks of gestation and is repeated in high-risk women who have had a negative result in the first test. (NICE Clinical, 2015). Regarding pharmacological treatment for GD, it has been shown that nutritional treatment is sufficient in 80% of patients with GD to reach the therapeutic goal. (American Diabetes Association, 2007). In addition, studies suggest that mild exercise such as a 20- to 45-min walk after eating decreases postprandial blood sugar in women with GD. (Hui et al., 2006). Therefore, pharmacological treatment should only be considered when diet and exercise do not achieve the target figures, in this case, both human rapid-acting insulin and its analogs lispro and aspart are considered safe since their effectiveness has been demonstrated and there are no reports of teratogenicity. (Langer, 2006; Metzger et al., 2007).

We highlight the link between the development of alterations in glucose metabolism during pregnancy as a preventable risk factor but that predisposes offspring to suffer CV events during their life.

Being exposed to high glucose concentration in the womb can lead to an increase in ROS and ER stress, a decrease in NO synthesis; thickening of the vessel wall, a decrease in the lumen of the vessel and damages in kidney´s functions and structure that can later on set the development of hypertension. Created by Biorender.

## Conclussion

This review confirms the relationship between HT/DM, focusing in hyperglycemia in pregnancy and the risk of CVD in adulthood; however, more studies need to be carried out to be able to define both the causality and the prevalence of the development of HT in the offspring of diabetic mothers, since there are many variations between populations, probably due to lifestyle, genetics, and other factors that remain to be defined.

## Limitations of the study

The review was done only with information in free full texts available in PubMed and Web of Science because we didn´t have the means to pay for articles. In the case of animal models, the main limitation of the STZ model is that the mechanism of action of the drug is the destruction of the beta cells of the pancreas and therefore there is no production of insulin, which would resemble type 1 diabetes. However, the progression of GD occurs due to the development of IR (type 2 diabetes). Although it is not the correct way to obtain high blood glucose values ​​in the mothers, what is ultimately important is to expose the offspring to hyperglycemia in their mothers’ wombs and then evaluate its consequences.
